# Case report: pulsed radiofrequency surgery combined with platelet-rich plasma injection in the treatment of supraspinatus injury

**DOI:** 10.1097/MD.0000000000027797

**Published:** 2021-12-23

**Authors:** Hui Jin, Yao Gao, Youbo Ji, Rui Xu, Hao Zuo, Zhonghan Wang

**Affiliations:** a Department of Pain, The Second Hospital of Jilin University, Changchun, PR China; b Department of Endocrinology, Shanghai National Research Center for Endocrine and Metabolic Disease, State Key Laboratory of Medical Genomics, Shanghai Institute for Endocrine and Metabolic Disease, Ruijin Hospital, Shanghai Jiaotong University School of Medicine, Shanghai, PR China; c Department of Orthopedics, The Second Hospital of Jilin University, Changchun, Jilin, PR China.

**Keywords:** platelet-rich plasma, radiofrequency, supraspinatus

## Abstract

**Rationale:**

The shoulder joint is the most movable joint of the human body, and the incidence of aseptic inflammation of the muscles and tendons around the shoulder joint and acute and chronic muscle injuries is relatively high. Pulsed radiofrequency neuromodulation technology is gradually being used in shoulder joint diseases. Platelet-rich plasma (PRP) is a high-power platelet plasma solution obtained by centrifugation of autologous blood. Platelet cells contain many growth factors that promote tissue repair.

**Patient concerns:**

Shoulder soreness, limited movement of the shoulder joint, abduction of the upper limbs, and aggravation of pain during flat lifting. The pain radiates to the deltoid muscle stop and forearm.

**Interventions:**

In this study, radiofrequency pulses combined with PRP were used to treat supraspinatus muscle injury and explore new methods for the treatment of shoulder joint muscle and tendon injuries represented by supraspinatus muscle injury.

**Diagnosis:**

We reported 4 patients with supraspinatus injury who received radiofrequency pulse combined with PRP treatment in our hospital.

**Outcomes:**

After treatment, the patients were followed up at the first month, the third month, and the sixth month, and the Constant–Murley shoulder score and visual analog scale were used to comprehensively evaluate the postoperative improvement of the patients. There was no significant increase in postoperative pain, the Constant–Murley shoulder Score was significantly increased, the range of movement of the shoulder joint was significantly improved, and there were no postoperative complications.

**Lessons:**

The combined application of the 2 treatments can make full use of the analgesic effect of pulsed radiofrequency technology and the repairing effect of PRP, and can maximize the advantages of the 2 more advanced treatment methods in the field of minimally invasive.

## 1 Introduction

The shoulder joint is the most movable joint of the human body. The incidence of aseptic inflammation of the muscles and tendons around the shoulder joint and acute and chronic muscle injury is relatively high.^[1]^ According to reports in the literature, the incidence can reach more than 20%.^[2]^ The supraspinatus muscle starts from the supraspinatus fossa of the scapula, and the tendon passes through the narrow space under the coracoid acromion ligament and subacromial capsule, and above the shoulder joint capsule, and ends at the upper part of the greater tuberosity of the humerus. Its function is to fix the humerus on the scapula. The glenoid, and coordinated action with the deltoid muscle to make the upper limbs abduct, due to frequent activities and the intersection of shoulder muscle contraction force, it is easy to damage.^[3,4]^ At present, most conservative treatments are adopted in clinical treatment, including physical therapy, oral nonsteroidal anti-inflammatory drugs, local closed injections, etc. There is no significant difference between their therapeutic effectiveness and surgical treatment.^[5,6]^ The treatment methods are constantly innovating and improving, but there is still a lack of unified standards and understanding, and each treatment has different advantages, disadvantages and indications.^[7]^

In recent years, with the development of minimally invasive treatment technology and regenerative medicine, the treatment of supraspinatus injury has made great progress.^[8]^ Pulsed radiofrequency neuromodulation technology has gradually been applied in shoulder joint diseases. The principle of pulsed radiofrequency technology treatment^[9]^: stimulate the plasticity of the central pain pathway that processes the incoming pain signal, such as activating the neurons in the superficial layer of the posterior horn; activate the spinal cord inhibitory mechanism; similar to the effect of local electromagnetic field, it changes the blood flow and neurotrophic of nerve fiber myelin cells; adjusted the content of pain mediators in the central nervous system, such as substance P and endorphins; and the local heating of the electric current loosens the adhesion material around the nerve and changes the pathological nerve coupling phenomenon.

Platelet-rich plasma (PRP) technology uses autologous blood centrifugation to obtain high-power platelet plasma solution in the treatment of shoulder muscle injuries.^[10]^ Platelet cells contain many growth factors and cytokines that promote tissue repair.^[11]^ It has been confirmed that PRP is rich in 7 major protein growth factors secreted by platelets, including 3 platelet-like growth factors (PDGFαα, PDGFαβ, and PDGFββ), 2 transforming growth factors 1 and 2, and vascular endothelial growth factor and epithelial growth factor.^[12]^ In addition, the PRP mixture also contains other biologically active factors (such as serotonin histamine, dopamine, calcium ions, adenylate) and cell adhesion molecules (fibronectin). After tissue injury, platelets will immediately migrate and aggregate to the injured area, secrete the above-mentioned factors, initiate the coagulation-inflammatory process, stimulate cell proliferation, chemotaxis, differentiation, blood vessel formation and regulate immune inflammatory response; Cell adhesion molecules such as fibronectin in platelet clots can promote cell migration and enhance the biological activity potential of PRP.^[13]^ The damage and repair of ligaments, tendons, cartilage and other tissues usually go through 3 stages: inflammation, cell proliferation, and remodeling. The activation of growth factors and cell factors runs through the entire process, connecting signals across membranes, and starting intracellular genes. Expression and production of new proteins regulate cell proliferation, chemotaxis, differentiation, outer matrix production and blood vessel formation; at the same time, the cytokines and biologically active substances released by PRP affect the basic biological metabolic processes of tissues. Therefore, PRP can promote the repair of tissue damage.^[14,15]^

## 2 Patient concerns

In this study, the patient presented with shoulder pain, limited movement of the shoulder joint, abduction of the upper limbs, and increased pain during flat lifting. The pain radiates to the deltoid muscle stop and forearm.

## 3 Diagnosis

A retrospective analysis of 4 patients (2 males and 2 females) in the Second Hospital of Jilin University from August 2019 to June 2020, with an average age of 52.3 years, all showed clear damage to the supraspinatus muscle (without complete rupture) by MRI. All patients have completed informed consent, know and agree to publish individual cases in journals. The patients were first divided into 0 to 3 grades according to the magnetic resonance performance: grade 0: normal rotator cuff; grade 1: tendinitis; grade 2: partial rupture of the tendon; grade 3: complete rupture of the tendon. The criteria for admission are: age 40 to 75 years; shoulder MRI is grade 2; and shoulder joint pain and mobility impairment symptoms last longer than 3 months.^[16]^ The exclusion criteria are: rotator cuff injury without shoulder joint pain only with MRI manifestations; full-thickness rotator cuff injury; shoulder joint osteoarthritis, rheumatoid arthritis, etc; and those who have a history of shoulder surgery and a history of mental illness. Before treatment, the patient's shoulder joint range of motion, muscle strength, pain score and other data were recorded in detail, and a trial nerve block was performed before the operation.

## 4 Interventions

### 4.1 Position

The patient adopts a forward neck sitting position, with his hands flexed on his thighs, and the patient feels comfortable in a comfortable position, fully exposing the lesion.

### 4.2 Skin marking

First draw the scapular spine on the skin of the patient's shoulder. Carefully check the tenderness points of the affected muscles and mark them on the skin.

### 4.3 Needle puncture

After routine disinfection, use 0.5% lidocaine 1 mL for local infiltration anesthesia from the skin, subcutaneous to the bone surface. Use a 10 cm long, 10 mm exposed tip of the radio frequency trocar to puncture the scapula and spinal bone vertically or diagonally at the skin mark, and draw back airless and bloodless.

### 4.4 B-guided puncture operation

**Figure 1:**
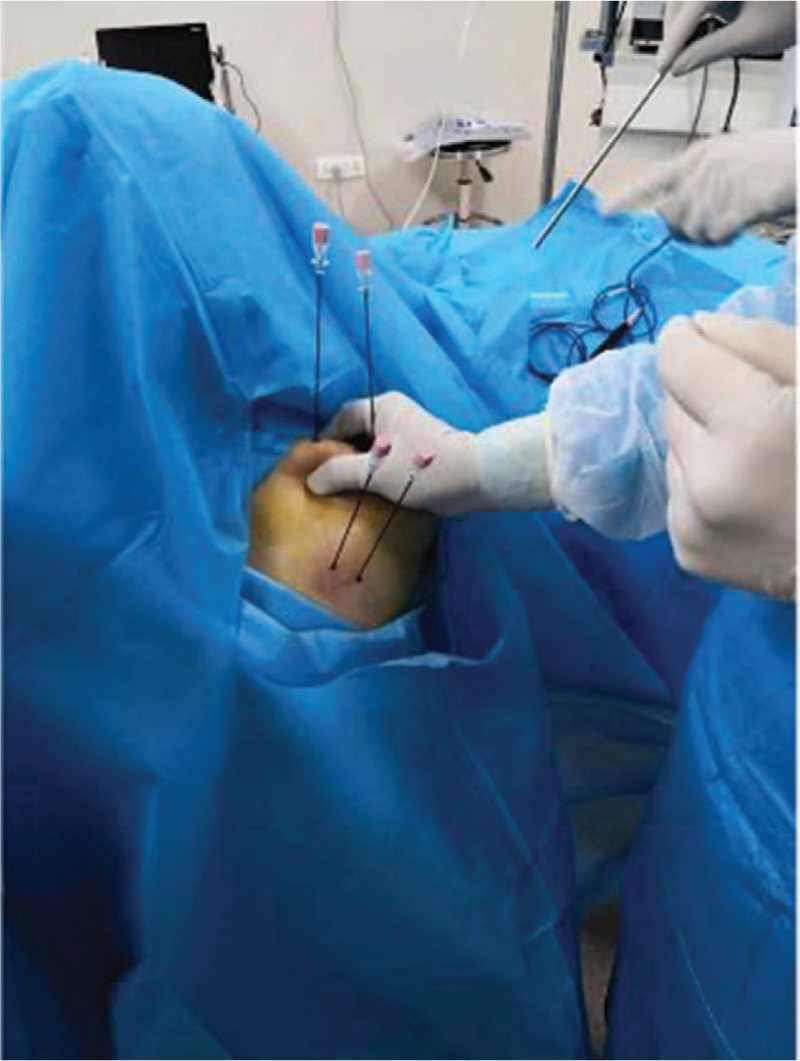
Shoulder joint radiofrequency treatment.

Because the supraspinatus muscle is close to the tip of the lung, accidental injury can cause pneumothorax to be dangerous. Therefore, the cases in this study were guided by B-ultrasound to clarify the position of the puncture needle and the greater tuberosity of the humerus, determine the attachment point of the supraspinatus muscle, and the depth of the pleura and lungs to avoid accidental injury. The appearance of the pleura and lungs is generally like “waves on the beach” under ultrasound.^[17]^ Instructing patients to take deep breaths can enhance imaging and resolution. During puncture, the patient takes a seated position and can use out-of-plane technology to locate the puncture point to be treated and mark it. The marked point is the puncture point. Place the marked point on the lower edge of the midpoint of the ultrasound probe to witness, and gradually adjust the needle trajectory, and start radiofrequency after the needle tip touches the bone (Fig. 1).

### 4.5 Pulse radio frequency

After the radio frequency needle is punctured in place, start the radio frequency pulse radio frequency to determine the impedance to be soft tissue, and maintain each point at 42°C for 120 seconds.

### 4.6 PRP preparation

**Figure 2:**
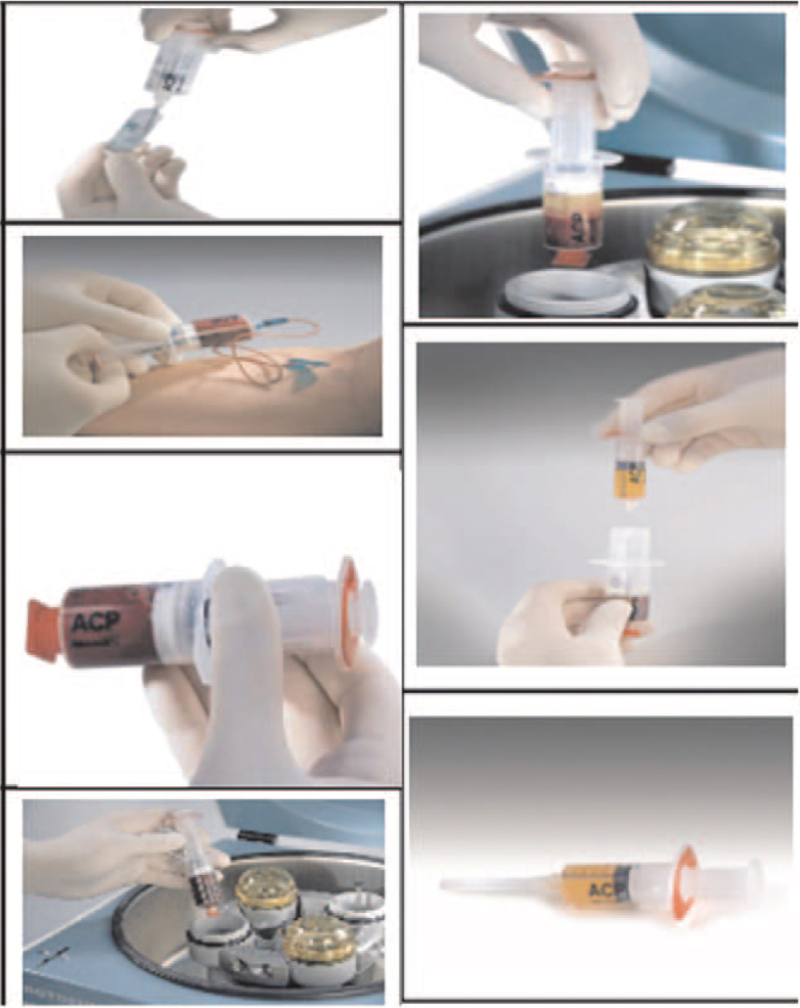
PRP production process.

While the pulsed radio frequency treatment is started, use the PRP preparation package (Shanghai Ruishi Medical Instrument Co., Ltd.) to draw 50 mL of venous blood, add 1 mL of anticoagulant, and discard the red blood cell suspension after centrifugation to obtain 4 to 5 mL of PRP solution, extract 1 mL calcium gluconate solution and add it to PRP (Fig. 2). After the pulsed radiofrequency treatment is over, PRP is injected into the supraspinatus muscle injury according to the mark.

### 4.7 Postoperative treatment

Twenty-four hours after the operation, the treatment area can be treated with microwave, ultrasound or polarized infrared heat rays. Anti-inflammatory and analgesic drugs were treated for 1 week.

## 5 Outcomes

There was no statistical difference in gender, side and age of the 4 patients. After treatment, they were followed up at 1 month, 3 months, and 6 months, and the Constant–Murley shoulder score and visual analog scale were used. Comprehensive evaluation of the patient's pain, daily activity ability, active range of activity, muscle strength and other aspects of improvement 6 months after surgery.

### 5.1 Clinical function evaluation

**Figure 3:**
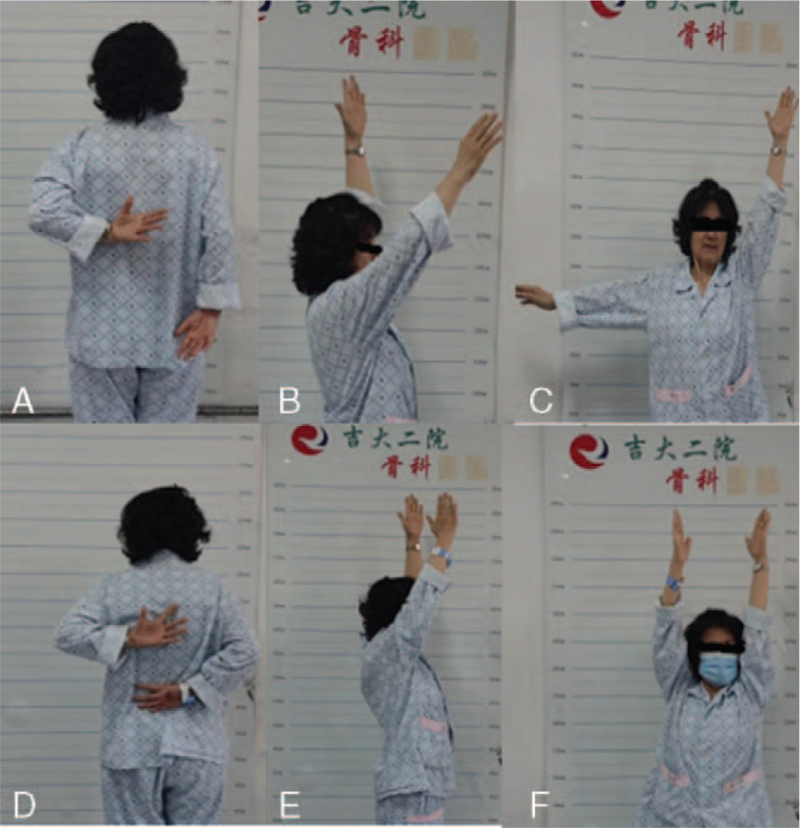
Comparison of shoulder joint range of motion before and after treatment (1 month of treatment). A. Internal rotation (preoperative) B. Forward bending (preoperative) C. Abduction (preoperative). D. Internal rotation (postoperative) E. Forward bending (postoperative) F. Abduction (postoperative).

The visual analog scale score of 4 patients decreased significantly at 1 month after operation, and there was no significant increase in pain at 3 and 6 months follow-up. The Constant–Murley shoulder score was significantly increased at 1 month after surgery, and there was no significant decrease in the score at the 3 and 6 months follow-up (Table 1); the active flexion, abduction, and internal rotation of the shoulder joint before and after surgery were significantly improved (Figs. 2 and 3).

**Table 1:** VAS and CMS scores of 4 patients.

Patients (No.)	VAS	CMS
No.1 Before surgery	9	45
Postoperative 1 months	3	78
Postoperative 3 months	3	80
Postoperative 6 months	2	79
No.2 Before surgery	8	49
Postoperative 1 months	3	82
Postoperative 3 months	2	80
Postoperative 6 months	2	81
No.3 Before surgery	9	42
Postoperative 1 months	4	78
Postoperative 3 months	3	77
Postoperative 6 months	2	79
No.4 Before surgery	9	46
Postoperative 1 months	3	77
Postoperative 3 months	2	75
Postoperative 6 months	1	77

CMS = Constant–Murley shoulder score, VAS = visual analog scale.

### 5.2 Postoperative complications

All patients had no complications such as wound swelling, exudation, fever, nerve injury, and vascular injury, and no antibiotics were used.

## 6 Discussion

It is the first time that pulsed radiofrequency technology combined with PRP technology has been used to treat supraspinatus injury, and there is no similar report. The principle of radiofrequency treatment is mainly to heat to degenerate and coagulate glycoproteins, inactivate the nerve endings at the stump of the rotator cuff, eliminate congestion and edema at the stump of the torn tissue, and reduce inflammation, thereby reducing pain and promoting healing.^[18,19]^ Previously, the prevailing view was that once the rotator cuff is torn, the tear will increase over time, and may cause the rotator cuff muscle to degenerate and cannot be repaired. As a result, the function of the shoulder joint is disabled, so early surgical treatment has been preferred in the past.^[20]^ In recent years, some scholars, through clinical research and analysis, have proposed that pulsed radiofrequency treatment has a good effect on mild to moderate supraspinatus tears, and there is no significant difference from surgical treatment; Long-term follow-up showed no significant change in the size of the tear, and no significant change in the supraspinatus fat infiltration, indicating that the torn rotator cuff has not undergone significant degeneration and is irreparable.^[21]^ The clinical application and research of PRP are progressing rapidly. Related reports pointed out that the role of PRP in the repair of rotator cuff injuries is to stimulate the healing of the tendon-bone interface and reduce pain and inflammation.^[22]^ In nonsurgical cases, the lack of repair factors between tendon-bone is the main reason why rotator cuff injuries are difficult to heal and the rate of re-tearing after surgery is high. PRP is a biological strategy to effectively promote tendon–bone healing. One of the most studied adjuvants for rotator cuff tear repair.^[23]^ Recent studies have evaluated the bio-enhancement function of PRP in the healing process. PRP can produce collagen, growth factors, and may increase the number of available stem cells, thereby providing a high concentration of α-particles containing biologically active parts to the area of soft tissue damage.^[24]^ Rotator cuff injury repair is a process of inflammation, repair, and remodeling. Many growth factors released by autocrine and paracrine promote cell proliferation and matrix deposition in the repair stage. These growth factors include basal fibroblast growth factor, vascular endothelial growth factor, platelet-derived growth factor, transforming growth factor β and insulin-like growth factor.^[25]^ The attraction of PRP is the combination or concentration of growth factors, because the result of rotator cuff injury repair is to restart scar formation rather than histologically normal insertion site. Therefore, the addition of these growth factors can biologically enhance the repair site. Clinical trials have proven that PRP can promote the remodeling of blood vessels in damaged parts and accelerate the repair of tendons. A new type of treatment such as PRP may be a better choice for the treatment of this type of disease.^[26]^

## 7 Conclusion

The purpose of the combined application of the 2 treatments is to make full use of the analgesic effect of pulsed radiofrequency technology and the repairing effect of PRP, which can maximize the advantages of the 2 more advanced treatment methods in the field of minimally invasive. Radiofrequency treatment uses heating to coagulate protein, inactivate nerve endings, and eliminate edema, so the analgesic effect is better, but the repairing effect is not stronger than other conservative treatments. The principle of PRP treatment is to produce collagen and growth factors, increase the number of stem cells locally, and provide bioactive particles with powerful repair functions. The 2 can better promote the improvement of symptoms of periarthritis and rotator cuff injury and shorten the treatment course, so that patients can restore shoulder joint function in the shortest time and return to society as soon as possible.

## Author contributions

**Conceptualization:** Hui Jin, Yao Gao, Youbo Ji, Rui Xu, Hao Zuo, Zhonghan Wang.

**Data curation:** Hui Jin, Yao Gao, Youbo Ji, Rui Xu, Hao Zuo, Zhonghan Wang.

**Formal analysis:** Hui Jin, Yao Gao, Youbo Ji, Rui Xu, Hao Zuo, Zhonghan Wang.

**Funding acquisition:** Hui Jin, Yao Gao, Youbo Ji, Rui Xu, Hao Zuo, Zhonghan Wang.

**Investigation:** Hui Jin, Yao Gao, Youbo Ji, Rui Xu, Hao Zuo, Zhonghan Wang.

**Methodology:** Hui Jin, Yao Gao, Youbo Ji, Rui Xu, Hao Zuo, Zhonghan Wang.

**Project administration:** Hui Jin, Yao Gao, Youbo Ji, Rui Xu, Hao Zuo, Zhonghan Wang.

**Resources:** Hui Jin, Yao Gao, Youbo Ji, Rui Xu, Hao Zuo, Zhonghan Wang.

**Software:** Hui Jin, Yao Gao, Youbo Ji, Rui Xu, Hao Zuo, Zhonghan Wang.

**Supervision:** Hui Jin, Yao Gao, Rui Xu, Hao Zuo, Zhonghan Wang.

**Validation:** Hui Jin, Yao Gao, Rui Xu, Hao Zuo, Zhonghan Wang.

**Visualization:** Hui Jin, Yao Gao, Rui Xu, Hao Zuo, Zhonghan Wang.

**Writing – original draft:** Hui Jin, Yao Gao, Rui Xu, Hao Zuo, Zhonghan Wang.

**Writing – review & editing:** Hui Jin, Yao Gao, Rui Xu, Zhonghan Wang.
